# Stage 2 Registered Report: Anomalous perception in a Ganzfeld condition - A meta-analysis of more than 40 years investigation

**DOI:** 10.12688/f1000research.51746.3

**Published:** 2024-02-08

**Authors:** Patrizio E. Tressoldi, Lance Storm

**Affiliations:** 1 Studium Patavinum, University of Padua, Padova, Italy; 2 School of Psychology, University of Adelaide, Adelaide, Australia

**Keywords:** Rgistered Report, meta-analysis, ganzfeld, anomalous perception

## Abstract

This meta-analysis is an investigation into anomalous perception (i.e., conscious identification of information without any conventional sensorial means). The technique used for eliciting an effect is the Ganzfeld condition (a form of sensory homogenization that eliminates distracting peripheral noise). The database consists of studies published between January 1974 and December 2020 inclusive.

The overall effect size estimated both with a frequentist and a Bayesian random-effect model, were in close agreement yielding an effect size of approximately .08 (.04 -.12). This result passed four publication bias tests and seems not contaminated by questionable research practices.

Trend analysis carried out with a cumulative meta-analysis and a meta-regression model with year of publication as a covariate, did not indicate sign of decline of this effect size.

The moderators’ analyses show that the selected participants’ effect size was almost three-times that obtained by non-selected participants and that tasks that simulate telepathic communication show a two-fold effect size for tasks requiring the participants to guess a target.

The Stage 1 Registered Report can be accessed here: https://doi.org/10.12688/f1000research.24868.3

## Introduction

The possibility of identifying pictures or video clips without conventional (sensorial) means, in a ganzfeld environment, is a decades old controversy, dating back to the pioneering investigation of Charles Honorton, William Braud and Adrian Parker between 1974 and 1975 ( Parker, 2017).

In the prototypical procedure, the participant (as percipient) is tested in a room isolated from external sounds and visual information. After he/she is made comfortable in a reclining armchair, he/she receives instructions related to the task during the ganzfeld condition. Even if there are different verbatim versions, the instructions describe what the percipient should do mentally in order to detect the information related to the target and how to filter out the mental contents not related to it. This information is described aloud and recorded for playback before or during the target identification phase. After a relaxation phase, which can range from 5 to 15 minutes, the percipient is exposed to the ganzfeld condition for a period ranging from 15 to 30 minutes.

In the ganzfeld environment, a German term meaning ‘whole field’, participants are immersed in a homogeneous sensorial field where peripheral visual information is masked out by red light diffused by translucent hemispheres (often split halves of ping-pong balls or special glasses) placed over the eyes, while a relaxing rhythmic sound, or white or pink noise, is fed through headphones to shield out peripheral auditory information.

Once the participant is sensorially isolated from external visual and auditory stimulation, he/she is thought to be in a favorable condition for producing inner mental contents about a randomly-selected target hidden among some decoys, usually three or four.

During this phase, the participant describes verbally all images, feelings, and emotions, they deem related to the target, which is usually a picture or a short video-clip of real objects or events.

The mentation (verbal report) produced by the participant can either be used to guide his/her target selection, or it can be used by the judge to assist in an independent judging process.

A variant of the judgment phase is to send the recording of the information retrieved during the ganzfeld phase to an external judge for independent ratings of the target. In order to prevent voluntary or involuntary leakage of information about the target by the experimenters, the research assistant who interacts with the participants must be blind to the target identity until the participants’ rating task is over.

The choice of the target and the decoys is usually made using automatic random procedures, and scores are automatically fed onto a scoring sheet.

There are three different ganzfeld conditions:

• Type 1 (Precogniyion): the target is chosen after the judgment phase;
• Type 2 (Clayrvoyance): the target is chosen before the ganzfeld phase;
• Type 3 (Telepathy): the target is chosen before the ganzfeld phase and presented to a partner of the participant isolated in a separate and distant room. From an historical perspective, this last type is considered the typical condition.

These differences are related to some theoretical and perceptual concepts we will discuss later. It is important to note that type of task makes no difference to the participant who only engages in target identification *after* the ganzfeld phase.

### Review of the Ganzfeld Meta-Analyses

It is interesting to note that most of the cumulative findings (meta-analyses) of this line of investigation were periodically published in the journal *Psychological Bulletin.*

Honorton (1985) undertook one of the first meta-analyses of the many ganzfeld studies completed by the mid-1980s. In total, 28 studies yielded a collective hit rate (correct identification) of 38%, where mean chance expectation (MCE) was 25%. Various flaws in his approach were pointed out by Hyman (1985), but in their joint-communiqué they agree that “there is an overall significant effect in this database that cannot reasonably be explained by selective reporting or multiple analysis” ( Hyman & Honorton, 1986, p. 351).

A second major meta-analysis on a set of ‘autoganzfeld’ studies was performed by Bem & Honorton (1994). These studies followed the guidelines laid down by Hyman & Honorton (1986). Moreover, the autoganzfeld procedure avoided methodological flaws by using a computer-controlled target randomization, selection, and judging technique. The overall reported hit rate of 32.2% exceeded again the mean chance expectation.

Milton & Wiseman (1999) meta-analyzed further 30 studies collected for the period 1987 to 1997, reporting an overall nonsignificant standardized effect size of 0.013. However, Jessica Utts (personal communication, December 11, 2009) using the exact binomial test on trial counts only ( *N* = 1198; Hits = 327), found a significant hit rate of 27% ( *p* = 0.036).

Storm & Ertel (2001) comparing Milton & Wiseman’s (1999) database with Bem & Honorton’s (1994) one, found the two did not differ significantly. Furthermore, Storm and Ertel went on to compile a 79-study database, which had a statistically significant average standardized effect size of 0.138.

Storm *et al.* (2010), meta-analyzed a database of 29 ganzfeld studies published during the period 1997 to 2008, yielding an average standardized effect size of 0.14. Rouder *et al.* (2013) reassessing Storm *et al.*’s (2010) meta-analysis with a Bayesian approach, found evidence for the existence of an anomalous perception in the original dataset observing a Bayes Factor of 330 in support of the alternative hypothesis (p. 241). However, they contended the effect could be due to “difficulties in randomization” (p. 241), arguing that ganzfeld studies with computerized randomization had smaller effects than those with manual randomization. The reanalysis by Storm *et al.*’s (2013) showed that this conclusion was unconvincing as it was based on Rouder *et al.*’s faulty inclusion of different categories of study.

In the last meta-analysis by Storm & Tressoldi (2020), related to the studies published from 2008 to 2018, the overall standardized effect size was 0.133; 95%CI: 0.06-0.18.

### This study

The main aim of this study is to meta-analyze all available ganzfeld studies dating from 1974 up to December 2020 in order to assess the average effect size of the database with the more advanced statistical procedures that should overcome the limitations of the previous meta-analyses, e.g., the use of a common instead of a random model to take in account studies experimental designs heterogeneity, and the controls of publication bias. Furthermore, we aim to identify whether there are moderator variables that affect task performance. In particular, we hypothesize that participant type and type of task are two major moderators of effect size (see Methods section).

## Methods

### Reporting guidelines

This study follows the guidelines of the APA Meta-Analysis Reporting Standard ( Appelbaum *et al.*, 2018) and the Preferred Reporting Items for Systematic Review and Meta-Analysis Protocols ( PRISMA-P, Moher *et al.*, 2015).

All following analyses have been approved in the Stage 1 of this Registered Report ( Tressoldi & Storm, 2021). Supplementary and new analyses not approved in the Stage 1, are reported in the *Exploratory analyses* section in the Results.

### Studies retrieval

Retrieval of studies related to anomalous perception in a Ganzfeld environment is simplified, firstly by the fact that most of these studies have already been retrieved for previous meta-analyses, as cited in the introduction. Secondly, this line of investigation is carried out by a small community of researchers. Thirdly, most of the studies of interest to us are published in specialized journals that adopted the editorial policy of accepting papers with results that are statistically non-significant (according to the frequentist approach). This last condition is particularly relevant because it reduces the publication bias due to non-publication (file drawer effect) of studies with statistically non-significant results often as a consequence of a reduced statistical power.

Furthermore in order to integrate the previous retrieval method, we carried-out an online search with Google Scholar, PubMed and Scopus databases of all papers from 1974 to 2020 including in the title and/or the abstract the word “ganzfeld” (e.g., for PubMed: Search: ganzfeld [Title/Abstract] Filters: from 1974 – 2020).

### Studies inclusion criteria

The following inclusion criteria were adopted:

• Studies related to anomalous perception in a ganzfeld environment;
• Studies must use human participants only (not animals);
• Total number of participants in a study must be in excess of two to avoid the inherent problems that are typical in single case studies;
• Target selection must be randomized by using a true or a pseudo random algorithm Random Number Generator (RNG) in a computer or similar electronic device, or a table of random numbers.
• Randomization procedures must not be manipulated by the experimenter or participant;
• Studies must provide sufficient information (e.g., number of trials and outcomes) for the authors to calculate the direct hit-rates and effect size values, so that appropriate statistical tests can be conducted.
• Peer reviewed and not peer-reviewed studies e.g., published in proceedings excluding dissertations.

### Variables coding

For each included study, one of the authors, expert in meta-analyses, coded the following variables:

• Authors;
• Year of publication;
• Number of trials;
• Number of hits;
• Number of choices of each trial;
• Task type (Type 1,2 or 3);
• Participants type (selected vs. unselected). The authors of the study scored as ‘selected’ all participants that were screened for one or more particular characteristic deemed favourable for the performance in this type of task. All others were coded as ‘non-selected’
• Peer-Review level: Level = 1 for studies published in conference proceedings and *Researches In Parapsychology* (moderate peer-review); Level = 2, for the studies published in scientific journals with full peer-review.

The second author randomly checked all studies, and the data was compared with those extracted by the other author. Discrepancies were corrected by inspecting the original papers.

The complete database with all supporting information is available as *Underlying data* ( Tressoldi & Storm, 2020).

### Effect size measures

As standardized measure of effect size, we used the one applied in Storm, Tressoldi & Di Risio (2010) and Storm & Tressoldi (2020): binomial Z score/√number of trials, using the number of trials, the hits score and the chance probability as raw scores. The exact binomial Z score has been obtained applying the formula implemented online at http://vassarstats.net/binomialX.html. When this algorithm did not compute the *z* value when either number of trials or number of hits were low, we used the one-tailed exact binomial *p*-value, to find the inverse normal *z* by using the online app at http://www.fourmilab.ch/rpkp/experiments/analysis/zCalc.html where the formula of this conversion is described.

The standardized effect size was computed applying the formula Z/√N of trials.

As standard error, we used the formula: √(hit rate % * (1-hit rate %)/trials * chance proportion *(1-chance proportion)).

In order to take into account the effect size overestimation bias in small samples, the effect sizes and their standard errors, were transformed into the Hedge’s *g* effect sizes, with the corresponding standard errors by applying the formula presented in Borenstein *et al.* (2009, pp. 27–28: g = (1-(3/(4df-1)))* d)).

### Pooled estimate of the average effect

In order to account for the between-studies heterogeneity, the overall effect size estimation of the whole database has been calculated by applying both a frequentist and a Bayesian random-effect model for testing its robustness.

### Frequentist random-effect model

Following the recommendations of Langan *et al.* (2019), we used the restricted maximum likelihood (REML) approach to estimate the heterogeneity variance with the Knapp and Hartung method for adjustment to the standard errors of the estimated coefficients ( Rubio-Aparicio *et al.*, 2018).

Furthermore, in order to control for possible influence of outliers, we calculated the median and mode of the overall effect size applying the method suggested by Hartwig *et al.* (2020).

These calculations were implemented in the R statistical environment v.4.0.3 with the metafor package v. 2.4 ( Viechtbauer, 2017). See syntax details provided as extended data ( Tressoldi & Storm, 2020).

### Bayesian random-effect model

As priors for the average effect size we used a normal distribution with Mean = 0.1; *SD* = 0.03, constrained positive, lower bound = 0 ( Haaf & Rouder, 2020), given our expectation of a positive value. As prior for the tau parameter we used an inverse gamma distribution with shape = 1, scale = 0.15.

This Bayesian meta-analysis was conducted using the MetaBMA package v. 0.6.7 ( Heck *et al.*, 2017).

### Publication bias tests

Following the suggestions of Carter *et al.* (2019), we applied four tests to assess publication bias:

• the 3-parameter selection model (3PSM), as implemented by Coburn & Vevea (2019) with the package ‘ weightr’ v.2.0.2;
• the p-uniform* (star) v. 0.2.5 test as described by van Aert & van Assen (2019), and
• the sensitivity analysis using the Mathur & VanderWeele (2020) package PublicationBias v.2.3.0.
• The Robust Bayesian meta-analysis test implemented with the RoBMA package v.2.3.1 ( Bartoš & Maier, 2020).

The three parameters model represents the average true underlying effect, *δ*, the heterogeneity of the random effect sizes, τ ^2^ and the probability that there is a nonsignificant effect in the pool of effect sizes. The probability parameter is modelled by a step function with a single cut point at *p* = 0.025 (one-tailed), which corresponds to a two-tailed *p* value of 0.05. This cut point divides the range of possible *p* values into two bins: significant and nonsignificant. The three parameters are estimated using maximum likelihood ( Carter *et al.*, 2019, p. 124).

The *p*-uniform* test, is an extension and improvement of the *p*-uniform method. P-uniform* improves upon *p*-uniform giving a more efficient estimator avoiding the overestimation of effect size in case of between-study variance in true effect sizes, thus enabling estimation and testing for the presence of between-study variance in true effect sizes.

Sensitivity analysis, as implemented by Mathur & VanderWeele (2020), assumes a publication process such that “statistically significant” results are more likely to be published than negative or “nonsignificant” results by an unknown ratio, *η* (eta). Using inverse-probability weighting and robust estimation that accommodates non-normal true effects, small meta-analyses and clustering, it enables statements such as: “For publication bias to shift the observed point estimate to the null, ‘significant’ results would need to be at least 30-fold more likely to be published than negative or ‘non-significant’ results” (p. 1). Comparable statements can be made regarding shifting to a chosen non-null value or shifting the confidence interval.

The Robust Bayesian meta-analysis test is an extension of Bayesian meta-analysis obtained by adding selection models to account for publication bias. This allows model-averaging across a larger set of models, ones that assume publication bias and ones that do not. This test allows us to quantify evidence for the absence of publication bias estimated with a Bayes Factor. In our case we compared only two models, a random-effect model assuming no publication bias and a random-model assuming publication bias.

### Cumulative meta-analysis

In order to ascertain the overall trend of the cumulative evidence and in particular for testing the presence of a positive or negative trend effect, we performed a cumulative effect size estimation.

### Meta-regression

Furthermore, we estimated the overall effect size taking the variable “year of publication” as covariate using a meta-regression model.

### Moderators effects

We compared the influence of the following three moderators: (i) Type of participant, (ii) Type of task and (iii) Level of peer-review.

As described in the Variable Coding paragraph, the variable Type of participant, has been coded in a binary way: selected vs unselected. Type of task has been coded as Type 1, Type 2, and Type 3, as described in the Introduction and level of Peer-review as 1 for studies published in conference proceedings or 2, for the studies published in scientific journals with full peer-review.

### Statistical power

The overall statistical power was estimated using R package metameta v.0.1.1 ( Quintana, 2020). Furthermore, we calculated the number of trials necessary to achieve a statistical power of at least.80 with an α = .05. With this estimation we examined how many studies in the database reached this threshold.

## Results

**Figure 1.**
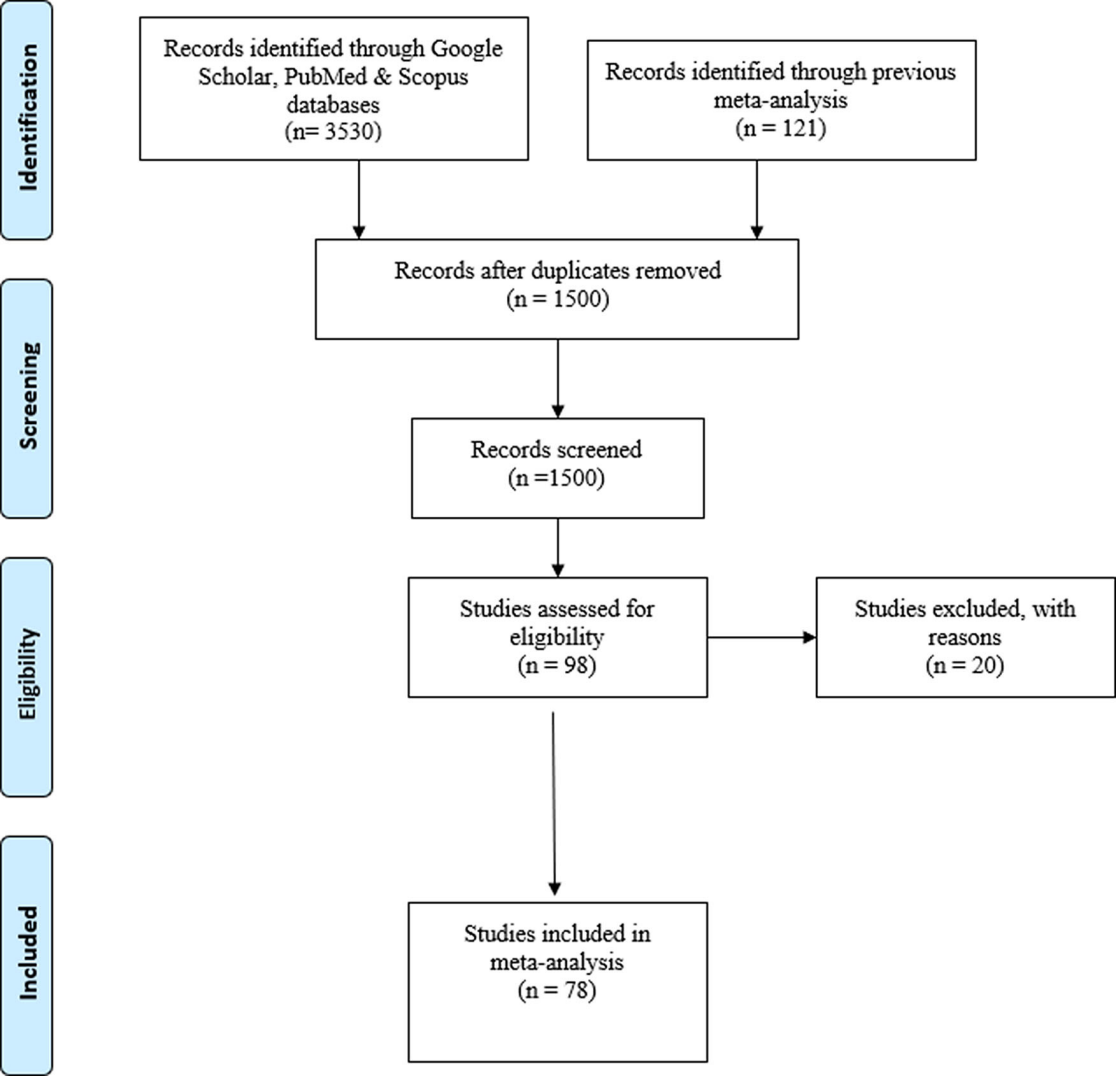
PRISMA flowchart.

The search and selection of the studies is presented in the PRISMA flowchart in Figure 1. As shown in the flowchart, our final database comprises 78 studies, for a total of 113 effect sizes carried out by 46 different principal investigators.

The list of all references related to the included and excluded studies is available in the GZMAReference List file and the data used for all the following statistical analyses is available in the GZMADatabase1974_2020 file in the *Underlying data* ( Tressoldi & Storm, 2020).

### Descriptive statistics

Descriptive statistics related to the variables trials, hits above chance, participants type, task types, peer-review level are presented in Table 1.

**Table 1.** Descriptive statistics of the main variables.

	Trials	Hits rate above chance	Task Type	Participants Type	Peer-review level
Mean ( *SD*)	42.8 (27.2)	.068 (.11) *			
Range	4-138	-.2 - .60			
Count (%)			Type 1: 5 (4.2) Type 2: 27 (23.8) Type 3: 81 (72)	Non-selected: 70 (62) Selected: 43 (38)	Level 1: 47 (41.6) Level 2: 66 (58.4)

* = this value is purely descriptive because not all studies are 4 free-choice designs.

*Comment:* The range of the number of trials as well as the hits percentage is quite wide. The number of task types show that the main types are Type 2: the target is chosen before the ganzfeld phase) and of Type 3: the target is chosen before the ganzfeld phase and presented to a partner of the participant isolated in a separate and distant room. Type 1 studies (target randomly selected after participant makes a choice) are only 5 (4.2%).

The percentage of studies using non-selected participants is greater (62% vs 38%) than that of studies using selected. Most studies (58.4%) were peer-reviewed.

### Pooled estimate of the average effect

The estimate of the average effect along with the corresponding 95% Confidence Intervals or Credible Intervals of both the frequentist and the Bayesian random-effect models as described in the Methods section, and values of τ ^2^ and I ^2^ ( Higgins & Thompson, 2002) with their confidence intervals, as measures of between-study variance, are presented in Table 2.

**Table 2.** Frequentist and Bayesian random-effect model results.

	Frequentist weighted ES (95% Confidence Intervals)	Bayesian weighted ES (95% Credible Intervals)	τ ^2^ (95% Confidence Intervals)	I ^2^ (95% Confidence Intervals)
Mean	.074 (.03 - .12)	.084 (.05 - .12)	.03 (.02 - .04)	63.8 (49.8 - 69.9)
P value or Bayes Factor _(H1/H0)_	.0009	89.5		

*Comment:* The frequentist and the Bayesian random-effect model parameters estimations are in close agreement, and both reject the null (H0) hypothesis with a high probability.

See also the reviewer 2 results based on the weighted least squares model.

In terms of hits percentage above chance, this small effect size corresponds to 6.8% (95%CIs: 4.7 – 8.9).

The level of heterogeneity is medium-large as expected by the influence of the moderators. Given this heterogeneity level, the values of the effect size median = .017 (-.025 - .06) and mode -.01 (-.13 - .10), are uninformative.

The forest plot is available as Figure S1 ( *Extended data* ( Tressoldi & Storm, 2020)).

### Outliers detection and influence

In order to detect the presence of influential outliers, we applied the “influence” function in the metafor package. These procedures identified two influential outliers. The results of the frequentist random-effect model without the influential outliers, are very similar to those with the outliers (mean ES: .078; 95% CIs: .03 - .12).

### Cumulative effect size

The results of the cumulative meta-analysis are represented with a cumulative forest plot in Figure S2 ( *Extended data* ( Tressoldi & Storm, 2020)). From the inspection of the cumulative forest plot, it emerges that the overall effect size stabilized around the cumulative evidence obtained up to 1997. Thus, it appears to be cumulative stable for more than 20 years.

### Meta-regression

The results of the meta-regression with “Year” as covariate, show a slope estimate of .0012 (95%CIs: -.002 - .005; *p* = .53).

*Comment:* These results support the hypothesis that the overall effect size is not affected by the year of publication of the experiments.

***Exploratory analyses***

Another way to observe the cumulative trend of the overall effect size, is to examine the evolution of the Bayes Factor and of Posterior Probability of H1 as the data accumulate. This information has been obtained using the option “sequential Bayes Factor” within the module Bayesian Meta-Analysis in the software JASP v.0.17.0 ( Jasp, 2020) that are presented in Figures S3 ( *Extended data* ( Tressoldi & Storm, 2020)). From these two plots it is possible to observe how the Bayes Factor started a positive linear trend after approximately 70 experiments. The maximum Posterior probability is achieved after approximately 80 experiments. The JASP file is available as *Underlying data* ( Tressoldi & Storm, 2020).

### Publication Bias tests

The results of the four publication bias tests described in the Methods section are presented in Table 3 and in the information that follows.

**Table 3.** Results of the p-uniform*, the 3-parameter selection model (3PSM), and the Robust publication bias tests.

	p-uniform*	3PSM	RoBMA
ES	.12	.15	.074
95% CIs	.04 - .21	.05 - .24	.00 - .12

The results of the Mathur & VanderWeele (2020) sensitivity analysis publication bias to shift the observed effect size point estimate to the .01 level, considered arbitrarily as the smallest effect size of interest, indicated that for publication bias to attenuate (to “explain away”) the observed overall effect size, affirmative results would need to be at least 20 fold more likely to be published than nonaffirmative results. See also reviewers’ 2 for further information about this test.

*Comment:* The overall effect size estimate passes all four publication bias tests.

### Moderators analyses

The weighted effect size along with the corresponding 95% confidence Intervals of the two types of participants, the three task types and the two peer-review level, are presented in Table 4.

**Table 4.** Effect sizes and 95% CIs related to the moderators’ categories.

	Selected Participants	Non-selected participants	Task Type 1	Task Type 2	Task Type 3	Peer-review level 1	Peer-review level 2
ES	.13	.04	.12	.04	.08	.073	.076
95% CIs	.06 - .20	-.01 - .09	-.08 - .33	-.04 - .13	.03 - .14	.01 - .13	.01 - .14

### Exploratory analysis

After looking at the participants selection and Task Type results, it was interesting to learn that selected participants and Task Type 3 combined, gave: *ES* = .14; 95%CIs: .06 - .22; not different from the results obtained by the selected participants in all three types of tasks.

*Comment*: Whereas it is clear that the levels of peer-review did not yield differences in the effect sizes, the selection of participants and the Task Types show substantial and statistically significant differences.

Selected participants show an almost three-fold increase in the effect size with respect to the non-selected participants. In terms of hits percentage above chance, this difference corresponds to 9.1%; 95%CI: 5-13 and 5.4%; 95%CI: 2.9 – 7.9, respectively.

Similarly, Tasks Type 1 and 3 show more than two-fold increase in ES compared to Type 2 tasks. However, the effect size observed with tasks Type 1, must be considered with caution given the low number of experiments (5).

### Statistical power

The median statistical power related to the observed overall effect size is .088. This result explains the fact that only 21 (18.5%) of the studies reported statistically significant results. For additional analyses related to the statistical power, see the reviewers’ 2 review.

## Discussion

The main aim of this meta-analysis was to get an overall picture of the evidence accumulated in more than 40 years of investigation related to an anomalous perception in a ganzfeld environment.

The main aim of this meta-analysis was to obtain an overall picture of the evidence accumulated over more than 40 years of investigation related to anomalous perception in a Ganzfeld environment.

The estimate of the average effect from 113 studies carried out from 1974 to June 2020 was small, but it turned out to be robust in both frequentist and Bayesian random-effect models.

As shown by the cumulative analysis and meta-regression with year of publication as covariate meta-analyses, this effect does not show a negative trend from 1974 to 2020 and is quite stable since 1997 and after 70-80 experiments.

Furthermore, the average effect passed four different publication bias tests, reducing the probability that it could be due to the selective reporting of studies with statistically significant results. This interpretation is also supported by the low number of studies (18.5%) with statistically significant results. This outcome is partly due to the practice of publishing statistically non-significant studies in specialized journals and proceedings related to this field of investigation.

Moreover, the similarity of effect size between the two levels of peer-review, add further support to the hypothesis that the “file drawer” is empty, that is that this meta-analysis includes all completed studies.

If we consider the average effect size, the lack of statistically significant results in many experiments is a consequence of their low statistical power, as shown by the very low median statistical power of the meta-analysis.

For those interested in this line of investigation the advice is clear. To achieve a statistical power of at least 0.80 with an alpha value of 0.05, each study must have at least 320 trials (estimated with G*Power, v.3.1.9.7, Faul, Erdfelder, Lang & Buchner, 2007).

However, this requirement can be reduced considerably if we consider the results of the moderators, in particular, the selection of participants and type of task. With selected participants carrying out a Type 3 task (i.e., with targets chosen before the ganzfeld phase and presented to a partner of the participant isolated in a separate and distant room simulating telepathic communication), the required trials can safely be reduced to 50.

Could the average results be contaminated using some questionable research practices ( John *et al.*, 2012), such as optional stopping, data exclusion, etc.? These practices were difficult to detect after the publication of the study, which is why it is recommended to register all methodological and statistical details before data collection. As far as it concerns this line of investigation, Wiseman, Watt and Kornbrot (2019), documented that preregistration was recommended well before the so-called replication crisis faced by most scientific fields. Furthermore, a simulation of the use of some questionable research practices carried out by Bierman, Spottiswoode, and Bijl (2016) on 78 studies related to anomalous perception in a Ganzfeld environment showed that even if the overall effect size could be inflated by the use of questionable research practices, it was not reduced to zero.

Even if this study is mainly devoted to the statistical analysis of the available evidence, it is important to consider possible theoretical frameworks that could account for such phenomena. Some of these are presented in the review by Cardeña (2018) and the book *Transcendent Mind* by Barušs and Mossbridge (2017). As a general theoretical framework, the main assumption is to consider the mind not derived or constrained by their biological correlates but ontologically independent from them, in agreement with some Western and Eastern philosophical interpretations, such as idealism ( Kastrup, 2018), dual-aspect monism ( Walach, 2020), Advaita Vedanta ( Sedlmeier & Srinivas, 2016), etc. If these interpretations of mind and consciousness are valid, what looks impossible or anomalous according to a physicalist or eliminative reductionist interpretation becomes perfectly normal.

## Summary and recommendations

The overall picture emerging from this meta-analysis is that there is sufficient evidence to claim that it is possible to observe a non conventional (anomalous) perception in a Ganzfeld environment. The available evidence does not seem to be contaminated by publication bias or questionable research practices. However, to increase the probability of detecting such phenomena, it is recommended to select participants and to use tasks that mimic telepathic communication.

As methodological advice, it is recommended that researchers preregister the methodological and statistical details in open access registries as proposed by Watt and Kennedy (2016) and others, or even better to use a registered report format that makes all procedures more transparent before and during data collection and analysis. One of the best examples to use as a model is the Transparent Psi Project ( Kekecs *et al.*, 2019).

We hope to update the evidence related to anomalous perception in a Ganzfeld environment with a meta-analysis of preregistered studies in the near future.

## Data availability

### Underlying data

Figshare: Registered Report - Anomalous perception in a Ganzfeld condition: A meta-analysis of more than 40 years investigation, https://doi.org/10.6084/m9.figshare.12674618.v13 ( Tressoldi & Storm, 2020).

This project contains the following underlying data:

- GZMADatabase1974_2020 (.jasp and.xlsx)
- GZMA Power (.csv)
- GZMA Reference List (.doc)

### Extended data

Figshare: Registered Report - Anomalous perception in a Ganzfeld condition: A meta-analysis of more than 40 years investigation, https://doi.org/10.6084/m9.figshare.12674618.v12 ( Tressoldi & Storm, 2020).

This project contains the following extended data:

- Syntax code for Stage 1 and Stage 2 Registered Report
- Figure S1: Forest Plot
- Figure S2: Cumulative Forest Plot
- Figure S3: Sequantial Bayes Factor

### Reporting guidelines

Figshare: PRISMA checklist for ‘Stage 2 Registered Report: Anomalous perception in a Ganzfeld condition - A meta-analysis of more than 40 years investigation’, https://doi.org/10.6084/m9.figshare.12674618.v12 ( Tressoldi & Storm, 2020).

Data are available under the terms of the Creative Commons Attribution 4.0 International license (CC-BY 4.0).
