# Supplementary material for: Stage 2 Registered Report: Anomalous perception in a Ganzfeld condition - A meta-analysis of more than 40 years investigation
Source: F1000Res. 2024 May 24;10:234. Originally published 2021 Mar 24. [Version 4] doi: 10.12688/f1000research.51746.4 (PMC11134153; doi:10.12688/f1000research.51746.4)
Supplement: Peer review history

# Reviewer report: Reviewer response for version 4

**DOI:** 10.5256/f1000research.166368.r282410

**Published:** 2024-05-28

**Authors:** Michel-Ange Amorim

**Affiliations:** 1 Universite Paris-Saclay, Gif-sur-Yvette, Île-de-France, France

The manuscript has been suitably revised and is an important contribution to the scientific literature on anomalous perception in the Ganzfeld.

Is the work clearly and accurately presented and does it cite the current literature?

Yes

If applicable, is the statistical analysis and its interpretation appropriate?

Partly

Are all the source data underlying the results available to ensure full reproducibility?

Partly

Is the study design appropriate and is the work technically sound?

Partly

Are the conclusions drawn adequately supported by the results?

Partly

Are sufficient details of methods and analysis provided to allow replication by others?

Partly

Reviewer Expertise:

psychophysics, cognitive psychology, cognitive and behavioral neuroscience, psi phenomena

I confirm that I have read this submission and believe that I have an appropriate level of expertise to confirm that it is of an acceptable scientific standard.

# Reviewer report: Reviewer response for version 3

**DOI:** 10.5256/f1000research.162122.r245034

**Published:** 2024-05-09

**Authors:** Michel-Ange Amorim

**Affiliations:** 1 Universite Paris-Saclay, Gif-sur-Yvette, Île-de-France, France

I appreciate the authors' responses and the effort made to revise their manuscript and the associated syntax code. Nevertheless, there are still some residual typos in the current version 3 of the manuscript, and even more damaging in an analysis (Publication Bias) that has been carried out.

**Introduction**

 "Type 1 (Precogniyion)" should be replaced by "Type 1 (Precognition)" ;

 and  "Type 2 (Clayrvoyance)" should be replaced by  "Type 2 (Clairvoyance)"

**Methods**

 About the formula, in "Effect size measures" section  :

 √(hit rate % * (1-hit rate %)/trials * chance proportion *(1-chance proportion))

 should be replaced by

 √( *hit rate proportion* * (1- *hit rate proportion*)/trials * *chance proportion* *(1- *chance proportion*)).

 or by 

 √( *hit rate* * (1- *hit rate*)/trials * *chance proportion* *(1- *chance proportion*)).

**Results**

 About the "Publication Bias tests" section of "Exploratory analyses" : "at least 20 fold more likely" should be replaced by "at least 4 fold more likely" 

 As illustrated in a separate *Jupyter Notebook* (R code and output) file (available through this link: PDF file2), I explain why the authors show re-run this analysis, possibly together with a complementary fail-safe analysis.

 This would allow authors to conclude that the findings suggest that although the meta-analysis is not immune to publication bias (given that a moderate level of bias favoring significant results in the published literature may account for the observed effect size), the meta-analysis results appear relatively robust against the potential impact of this bias as indicated by the high fail-safe N value.

Other Rmd problems :

 1) Although I could not test RoBMA in R (due to a problem with jags), the results of this analysis are in the GZMADatabase1974_2020.jasp  provided by the authors.

 2) The **Mode-based estimate** part of the Rmd code returned an error :

*> MBEMeta(beta.in, se.in, alpha=0.05,n_boot=1e4)*

*Error in density(beta.in, bw = bw, weights = weights) :*

*Argument 'x' must be an object of class "weightfunct".*

 However, when I ran the code that I previously modified (see my previous review) on the new data file, I obtained roughly similar results as the authors (a median of 0.016 where the authors found 0.017, and similar CIs)

**Discussion** 

 It's unclear to me why the "320 trials" estimate (using G*Power) stayed the same as in version 2 of the manuscript, in spite of the fact that the estimated overall ES changed from  .099 (.05-.14) to .08 (.04 -.12). 

 I also tried G*Power and found that for a two-tailed "Proportion: Sign test (binomial test)" with a ESg of 0.08, alpha = 0.05, and power = 0.80, the required sample size is N = 312.

 Could the authors be more specific about the type of analysis (parameters) they used in G*Power ?

Is the work clearly and accurately presented and does it cite the current literature?

Yes

If applicable, is the statistical analysis and its interpretation appropriate?

Partly

Are all the source data underlying the results available to ensure full reproducibility?

Partly

Is the study design appropriate and is the work technically sound?

Partly

Are the conclusions drawn adequately supported by the results?

Partly

Are sufficient details of methods and analysis provided to allow replication by others?

Partly

Reviewer Expertise:

psychophysics, cognitive psychology, cognitive and behavioral neuroscience, psi phenomena

I confirm that I have read this submission and believe that I have an appropriate level of expertise to confirm that it is of an acceptable scientific standard, however I have significant reservations, as outlined above.

# Response

**Published:** 2024-05-10

**Authors:** Patrizio Tressoldi

**Affiliations:** Studium Patavinum - Padova University, Italy

Thank you for your continuous accurate and constructive reviews we now have acknowledged in this new version.

 Here are replies to all your comments and suggestions:

**Introduction**

 "Type 1 (Precogniyion)" should be replaced by "Type 1 (Precognition)" ;

 and  "Type 2 (Clayrvoyance)" should be replaced by  "Type 2 (Clairvoyance)"

**Methods**

 About the formula, in "Effect size measures" section  :

 √(hit rate % * (1-hit rate %)/trials * chance proportion *(1-chance proportion))

 should be replaced by

 √( *hit rate proportion* * (1- *hit rate proportion*)/trials * *chance proportion* *(1- *chance proportion*)).

 or by 

 √( *hit rate* * (1- *hit rate*)/trials * *chance proportion* *(1- *chance proportion*)).

**Reply: corrected as suggested**

 Results

 About the "Publication Bias tests" section of "Exploratory analyses" : "at least 20 fold more likely" should be replaced by "at least 4 fold more likely" 

**Reply: corrected as suggested**

 As illustrated in a separate *Jupyter Notebook* (R code and output) file (available through this link: PDF file2 ), I explain why the authors show re-run this analysis, possibly together with a complementary fail-safe analysis.

 This would allow authors to conclude that the findings suggest that although the meta-analysis is not immune to publication bias (given that a moderate level of bias favoring significant results in the published literature may account for the observed effect size), the meta-analysis results appear relatively robust against the potential impact of this bias as indicated by the high fail-safe N value.

**Reply: we added the suggested text in the Discussion section. We didn’t add the Rosenthal fail-safe test given its limitations (Jin, Z. C., Zhou, X. H., & He, J. (2015). Statistical methods for dealing with publication bias in meta‐analysis.** ***Statistics in Medicine*** **,** ***34*** **(2), 343-360.)**

Other Rmd problems :

 1) Although I could not test RoBMA in R (due to a problem with jags), the results of this analysis are in the GZMADatabase1974_2020.jasp  provided by the authors.

 2) The **Mode-based estimate** part of the Rmd code returned an error :

*> MBEMeta(beta.in, se.in, alpha=0.05,n_boot=1e4)*

*Error in density(beta.in, bw = bw, weights = weights) :*

*Argument 'x' must be an object of class "weightfunct".*

 However, when I ran the code that I previously modified (see my previous review) on the new data file, I obtained roughly similar results as the authors (a median of 0.016 where the authors found 0.017, and similar CIs)

**Reply: happy you confirmed our results**

 Discussion 

 It's unclear to me why the "320 trials" estimate (using G*Power) stayed the same as in version 2 of the manuscript, in spite of the fact that the estimated overall ES changed from  .099 (.05-.14) to .08 (.04 -.12). 

 I also tried G*Power and found that for a two-tailed "Proportion: Sign test (binomial test)" with a ESg of 0.08, alpha = 0.05, and power = 0.80, the required sample size is N = 312.

 Could the authors be more specific about the type of analysis (parameters) they used in G*Power ?

**Reply: we estimated a one-tailed alpha = 0.05, given that only positive outcomes are related to anomalous perception**

# Reviewer report: Reviewer response for version 3

**DOI:** 10.5256/f1000research.162122.r245035

**Published:** 2024-03-09

**Authors:** Dean Radin

**Affiliations:** 1 Research, Institute of Noetic Sciences, Petaluma, California, USA; 2 Integral and Transpersonal Psychology, California Institute of Integral Studies, San Francisco, California, USA

This is acceptable. I see some typos (e.g., clairvoyance and precognition are misspelled), but otherwise it is okay.

Is the work clearly and accurately presented and does it cite the current literature?

Partly

If applicable, is the statistical analysis and its interpretation appropriate?

Yes

Are all the source data underlying the results available to ensure full reproducibility?

Yes

Is the study design appropriate and is the work technically sound?

Yes

Are the conclusions drawn adequately supported by the results?

Yes

Are sufficient details of methods and analysis provided to allow replication by others?

Partly

Reviewer Expertise:

Experimental psychology, parapsychology, psychophysiology, physics, and statistics.

I confirm that I have read this submission and believe that I have an appropriate level of expertise to confirm that it is of an acceptable scientific standard.

# Reviewer report: Reviewer response for version 3

**DOI:** 10.5256/f1000research.162122.r245036

**Published:** 2024-02-21

**Authors:** Pavo Orepic

**Affiliations:** 1 University of Geneva, Geneva, Switzerland

The authors did not address my concerns. I would suggest not approving this manuscript. My status is "Not Approved".

Is the work clearly and accurately presented and does it cite the current literature?

No

If applicable, is the statistical analysis and its interpretation appropriate?

Partly

Are all the source data underlying the results available to ensure full reproducibility?

Partly

Is the study design appropriate and is the work technically sound?

No

Are the conclusions drawn adequately supported by the results?

No

Are sufficient details of methods and analysis provided to allow replication by others?

Partly

Reviewer Expertise:

neuroscience, hallucination engineering, speech, inner speech, self-consciousness, self-voice perception, EEG, intracranial EEG

I confirm that I have read this submission and believe that I have an appropriate level of expertise to state that I do not consider it to be of an acceptable scientific standard, for reasons outlined above.

# Reviewer report: Reviewer response for version 2

**DOI:** 10.5256/f1000research.143962.r224487

**Published:** 2024-01-25

**Authors:** Pavo Orepic

**Affiliations:** 1 University of Geneva, Geneva, Switzerland

This is a meta-analysis of the studies investigating the Ganzfeld effect. It builds up on the previous meta-analyses by including the most recent studies and applying different, more detailed statistical approaches. I have several major concerns related to this work.

 My biggest concern is that I found the title (as well as the story) misleading. The paper is actually about telepathic communication through the Ganzfeld effect, and not the Ganzfeld effect itself. This research question is not clearly stated. The ganzfeld effect is a much wider phenomenon that is based on Gestalt psychology, which was not even introduced. Moreover, other Ganzfeld effect studies that investigated the phenomenon itself and its neural correlates (e.g. works of Jiri Wackermann or Timo Schmidt) besides its alleged use for telepathic communication were not mentioned.  Generally, the manuscript is quite difficult to follow, needs more thorough proofreading (as detailed by the other reviewer), and important information about the reviewed studies (specified below) is missing. I would suggest rewriting the manuscript such that its real purpose becomes clearer.

 Another major issue that makes the results unconvincing is the inclusion of studies that are not properly peer-reviewed. Using “peer review level” as a correlate or a covariate in analyses and not observing a difference between “proper” and “improper” actually raises more doubts about the quality of the “proper” studies.

 The Ganzfeld procedure itself (especially limited to this context of communication) is not clearly introduced. What is the “target”, the “decoys”, the “judging process”, etc? What are the instructions for the participants? How is the hit rate estimated and computed? The difference between the three different conditions is also not clear. How can a target be chosen after the judgment phase? A schematic would be useful. Some of the covered studies should be introduced in detail to give the reader the impression of what is actually investigated here. I only understood what the paper was about after I read another paper on the topic.

 A table summarizing the reviewed studies and their main approaches and findings is missing. Since the goal of the article also seems to be to contrast this meta-analysis with the previous ones, this table should also indicate which studies were indicated in which other meta-analyses.

 I find it also strange to use bulletpoints in such a way throughout the manuscript, especially in the Introduction. It breaks the flow of the narrative, which is largely missing in the first place. The paragraphs are often not connected – the manuscript reads more as a list than as a story.

 What is “autoganzfeld”? The difference between the introduced meta-analyses is not very clear. Did they cover different studies? What were their inclusion criteria and metrics? How is this meta-analysis advancing the previous ones – is it simply margining the data?

 I am also not convinced that the problem of publication bias is solved. I would like to see the Test for Excess Success (TES) which examines whether the reported success rate of a set of experiments agrees with the estimated magnitude of the effect. For a relevant discussion, see Chapter 10 from the book of Herzog et al., Understanding Statistics and Experimental Design, Learning Materials in Biosciences, https://doi.org/10.1007/978-3-030-03499-3_10). They describe an interesting scenario in which out of 10 papers investigating precognition, 9 found a significant result (90%), whereas TES indicated that there is a 6% chance of having the same degree of success as the original report.

 Even if the evidence for the telepathic phenomena proves to be true – there should be more discussion (as well as more introduction) on the supposed/proposed mechanisms behind it. What do different authors propose to explain the effect? How is this addressed in different studies? How in different meta-analyses? Are differences in supposed mechanisms taken into account? For a person completely outside of the field, such a “gentle introduction” is necessary to at least try to consider the possibility of such effects. I would suggest moving (and extending) the one paragraph talking about this from the Discussion to the Introduction, and in the Discussion focus on how the differences are reflected in different studies and meta-analyses.

 Generally, even though I find the topic very interesting, I do not find the work convincing. I am not familiar with the literature nor the methods in parapsychology, but for the field to advance and be more accepted by the scientific community, it would be useful if the research practices and writing style were done such that it is understandable to a person outside the field.

Is the work clearly and accurately presented and does it cite the current literature?

No

If applicable, is the statistical analysis and its interpretation appropriate?

Partly

Are all the source data underlying the results available to ensure full reproducibility?

Partly

Is the study design appropriate and is the work technically sound?

No

Are the conclusions drawn adequately supported by the results?

No

Are sufficient details of methods and analysis provided to allow replication by others?

Partly

Reviewer Expertise:

neuroscience, hallucination engineering, speech, inner speech, self-consciousness, self-voice perception, EEG, intracranial EEG

I confirm that I have read this submission and believe that I have an appropriate level of expertise to state that I do not consider it to be of an acceptable scientific standard, for reasons outlined above.

## References

[1] : Understanding Statistics and Experimental Design, Learning Materials in Biosciences. *https://link.springer.com/chapter/10.1007/978-3-030-03499-3_10#citeas* .2019; https://doi.org/10.1007/978-3-030-03499-3_10 10.1007/978-3-030-03499-3_10

# Response

**Published:** 2024-01-31

**Authors:** Patrizio Tressoldi

**Affiliations:** Studium Patavinum - Padova University, Italy

My biggest concern is that I found the title (as well as the story) misleading. The paper is actually about telepathic communication through the Ganzfeld effect, and not the Ganzfeld effect itself. This research question is not clearly stated.

 Reply: The title indicates anomalous perception as tested using ganzfeld principles. In the revised introduction the reader wil see that “telepathic communication” is only one parapsychological modality tested (i.e., Type 3), as precognition (Type 1) and clairvoyance (Type 2) are not specifically identified and listed. These are nominal terms, so neutral terms (i.e, ‘type’) is preferred throughout the paper.

 The ganzfeld effect is a much wider phenomenon that is based on Gestalt psychology, which was not even introduced. Moreover, other Ganzfeld effect studies that investigated the phenomenon itself and its neural correlates (e.g. works of Jiri Wackermann or Timo Schmidt) besides its alleged use for telepathic communication were not mentioned.  Generally, the manuscript is quite difficult to follow, needs more thorough proofreading (as detailed by the other reviewer), and important information about the reviewed studies (specified below) is missing. I would suggest rewriting the manuscript such that its real purpose becomes clearer.

**Reply: acknowledged, but we believe we have been very clear about the specific use of ganzfeld in parapsychology. We feel the review, coupled with the information provided in the Introduction, makes it quite clear what the history of Ganzfeld psi testing was all about. Other than that, it is not clear to us what additional information Reviewer #3 would require.**

 Another major issue that makes the results unconvincing is the inclusion of studies that are not properly peer-reviewed. Using “peer review level” as a correlate or a covariate in analyses and not observing a difference between “proper” and “improper” actually raises more doubts about the quality of the “proper” studies.

**Reply: It is not a case of “Improper” studies being assessed. Clearly, studies that might be regarded as having been reviewed to an ‘adequate’ or ‘sufficient’ standard (level 1) only, could easily have passed full review (level 2) since we showed that the level of peer-review “did not yield differences in the effect sizes”. That is, we should expect that a sharper peer-review would translate as significantly lower effect sizes under the assumption that psi effects are the result of design flaws.**

 The Ganzfeld procedure itself (especially limited to this context of communication) is not clearly introduced. What is the “target”, the “decoys”, the “judging process”, etc? What are the instructions for the participants? How is the hit rate estimated and computed? The difference between the three different conditions is also not clear. How can a target be chosen after the judgment phase? A schematic would be useful. Some of the covered studies should be introduced in detail to give the reader the impression of what is actually investigated here. I only understood what the paper was about after I read another paper on the topic.

 A table summarizing the reviewed studies and their main approaches and findings is missing. Since the goal of the article also seems to be to contrast this meta-analysis with the previous ones, this table should also indicate which studies were indicated in which other meta-analyses.

 I find it also strange to use bulletpoints in such a way throughout the manuscript, especially in the Introduction. It breaks the flow of the narrative, which is largely missing in the first place. The paragraphs are often not connected – the manuscript reads more as a list than as a story.

**Reply: Some of these problems are now fixed, and we find the use of bulletpoints is expedient and convenient for the readers (many meta-analyses employ this formatting).**

 What is “autoganzfeld”? The difference between the introduced meta-analyses is not very clear. Did they cover different studies? What were their inclusion criteria and metrics? How is this meta-analysis advancing the previous ones – is it simply margining the data?

**Reply: “autoganzfeld” was defined in the Introduction under the heading, Review of the Ganzfeld Meta-Analysis. In that review, the sequencing of the studies across the years indicates gradual improvements in study designs.**

 I am also not convinced that the problem of publication bias is solved. I would like to see the Test for Excess Success (TES) which examines whether the reported success rate of a set of experiments agrees with the estimated magnitude of the effect. For a relevant discussion, see Chapter 10 from the book of Herzog et al., Understanding Statistics and Experimental Design, Learning Materials in Biosciences, https://doi.org/10.1007/978-3-030-03499-3_10). They describe an interesting scenario in which out of 10 papers investigating precognition, 9 found a significant result (90%), whereas TES indicated that there is a 6% chance of having the same degree of success as the original report.

**Reply: We could have conducted any number of tests on publication bias, and still not satisfied many readers/critics; we have conducted various such tests in our past meta-analytic papers and found similar results (e.g., see Storm, Tressoldi, & Di Risio, 2010). Furthermore, if you check the z values of all experiments included in the database you will notice that only 30 (26.5%) are statistically significant, confirming that there is not an excess of sucess.**

 Even if the evidence for the telepathic phenomena proves to be true – there should be more discussion (as well as more introduction) on the supposed/proposed mechanisms behind it. What do different authors propose to explain the effect? How is this addressed in different studies? How in different meta-analyses? Are differences in supposed mechanisms taken into account? For a person completely outside of the field, such a “gentle introduction” is necessary to at least try to consider the possibility of such effects. I would suggest moving (and extending) the one paragraph talking about this from the Discussion to the Introduction, and in the Discussion focus on how the differences are reflected in different studies and meta-analyses.

**Reply: we agree possible mechanisms of the psi process could be discussed but it is likely there is more than one process besides telepathy to be discussed (i.e., precognition and clairvoyance as well), and to introduce various theories would go beyond the parameters of our paper which is largely statistical.**

 Generally, even though I find the topic very interesting, I do not find the work convincing. I am not familiar with the literature nor the methods in parapsychology, but for the field to advance and be more accepted by the scientific community, it would be useful if the research practices and writing style were done such that it is understandable to a person outside the field.

**Reply: we are very sorry that this paper is not suitable for researchers not familiar with the literature, and the methods in parapsychology, and we add the characteristics of a Stage 2 meta-analysis Registered Report. Unfortunately, we cannot satisfy your request, unless we change completely the type of paper (see previous comment).**

# Reviewer report: Reviewer response for version 2

**DOI:** 10.5256/f1000research.143962.r177854

**Published:** 2024-01-24

**Authors:** Michel-Ange Amorim

**Affiliations:** 1 Universite Paris-Saclay, Gif-sur-Yvette, Île-de-France, France

My report is provided in a separate file available through this link: PDF file.

Is the work clearly and accurately presented and does it cite the current literature?

Yes

If applicable, is the statistical analysis and its interpretation appropriate?

Partly

Are all the source data underlying the results available to ensure full reproducibility?

Partly

Is the study design appropriate and is the work technically sound?

Partly

Are the conclusions drawn adequately supported by the results?

Partly

Are sufficient details of methods and analysis provided to allow replication by others?

Partly

Reviewer Expertise:

psychophysics, cognitive psychology, cognitive and behavioral neuroscience, psi phenomena

I confirm that I have read this submission and believe that I have an appropriate level of expertise to confirm that it is of an acceptable scientific standard, however I have significant reservations, as outlined above.

# Response

**Published:** 2024-01-31

**Authors:** Patrizio Tressoldi

**Affiliations:** Studium Patavinum - Padova University, Italy

Thank you for your accurate review in particular the reproducibility of our results using our database and code syntax.

 Here follow our replies to all your comments.

 Main comments:

 1-At the beginning of the “Effect size measures” section, the authors write “As a standardized measure of effect size, we used […] Binomial Z score/√number of trials using the number of trials, the hits score

 and the chance probability as raw scores.”  However, in the xlsx data file provided by the authors, the

 values in the ES column correspond to Z/√N, with N as the number of participants rather than the

 number of trials in the study.

**Reply: even if using trials or participants as a unit of analysis changes only some decimals of all results for this database, in this revision we used trials as this is the standard choice in all meta-analyses related to the topic investigated.**

 2-I also share the concerns expressed earlier by S Schmidt and J Utts about using webpages to compute exact binomial Z score values, either directly (with http://vassarstats.net/binomialX.html) or indirectly (with http://www.fourmilab.ch/rpkp/experiments/analysis/zCalc.html from the p value provided by binomialX.html).

**Reply: what is important is the correctness of the formulas and not where they are implemented. In our case, the choice of webpages could allow independent controls without using other software. In our case, the formulas are available on the websites. Thank you for pointing us the error related to the Z score of the third experiment.**

 3- Publication Bias tests

 ….On the one hand, I find this statement incomplete and misleading because it ignores the confidence

 interval around the meta-analytic pooled point estimate.

 Reply: we added “These results suggest that for publication bias to attenuate (to “explain away”) the observed point effect lower CI interval to the null, affirmative results would need to be at least 10 fold more likely to be published than nonaffirmative results”, in the revised version as suggested.

 4- Could the authors give a reference or spell out the practical (or theoretical) reasons for this

 statement: “the .01 level, considered the smallest effect size of interest”?

**Reply: This is an arbitrary cutoff close to the null (zero) level for this line of investigation. This level can change in other research fields.**

 Further comments

 1-Table 1 reports several descriptive values. The mean (SD) values in the Hit rate column are for 4 free-choice designs. However, the corresponding note at the bottom of Table “this value is purely

 descriptive because not all studies are 4 free-choice designs” is ambiguous;

**Reply: we changed the proportion of hits with the proportion of hits above the chance level a measure that takes into account the differences in the chance level in the different experiments.**

 2-The manuscript mentions several supplementary figures that are not currently available at the Figshare link the authors provided

**Reply: Sorry for that. Now we have uploaded the Forest, the cumulative and the  Sequential Bayes Factor in the Stage 2 Supplementary Figures.doc file in the figshare repository.**

 3- Unforfunately, the authors do not provide any funnel plot figure. I recommend the authors to use the

 metaviz R library to generate a funnel plot with power regions

**Reply: in the text of the revised version we invited the readers to read your review and in particular your power funnel plot.**

 4- Rationale of the statistical analysis and Discussion of the results

 Surprisingly, they never clearly wrote down (or maybe I missed something?) that the theoretical GZ effect size would be “Z /√N = 0.1”.

**Reply: the use of this prior in the Bayesian meta-analysis is an arbitrary choice based on the overall frequentist meta-analysis, and the previous meta-analyses e.g. Storm & Tressoldi, 2010.**

# Reviewer report: Reviewer response for version 2

**DOI:** 10.5256/f1000research.143962.r205990

**Published:** 2023-11-02

**Authors:** Dean Radin

**Affiliations:** 1 Research, Institute of Noetic Sciences, Petaluma, California, USA; 2 Integral and Transpersonal Psychology, California Institute of Integral Studies, San Francisco, California, USA

This is a follow-up to a previous meta-analysis of the ganzfeld condition experimental database. It uses the latest meta-analytic techniques and adds further evidence to the original conclusion.

 My primary comment about this document is that it would be difficult for those who are not already familiar with the relevant literature to fully understand what this experiment is about. For example, in the Introduction, participants are referred to as "they," but given that "they" is sometimes used a neutral gender term, we cannot tell if this entails one person or two, who is describing their impressions aloud, how that information is recorded, who judges that information against the target, how many targets and decoys are involved (we read four at one point, and binary at another), and so on.

 Because of such confusions, I think it would be much clearer if the various types of ganzfeld condition experiments that are being considered were defined upfront, and then state how many participants are involved in each kind of study, and exactly what each of their roles are. As currently written, some of this information is provided later in the Introduction, but by the time the reader gets to that description they will already be confused.

 Other phases that might be clarified:

 > Once participants are sensorially isolated from external visual and auditory stimulation, they are in a favorable condition

 I'd prefer a more cautious " *thought to be* a favorable condition"

 > in the mainstream journal *Psychological Bulletin.*

 The word *mainstream *is unnecessary.

 > Moreover the autoganzfeld procedure avoids

 Maintain consistency in tenses. Thus, "avoided" and not avoids.

 > They overall reported hit rate 

 You mean " *The *overall reported hit rate"

 > average standardized effect size

 Meaning? There are many definitions for effect size.

 > with the more advanced statistical procedures that should overcome the limitations of the previous meta-analyses

 Such as?  List these limitations.

 > have been approved in the Stage 1

 The Stage 1 what? That term only makes sense if one is already familiar with how this journal works. 

 > the editorial policy of accepting paper 

 You mean "papers".  Note: There are many other examples like this that a proofreader would catch. I suggest that the authors do a very careful review  of the text for these kinds of grammatical mistakes. I will mention some (not all) of them below.

 > Studies must use human participants only (not animals);

 Are there ganzfeld studies with animals?

 > Number of participants must be in excess of two to avoid the inherent problems that are typical in case studies;

 This is confusing because we don't know if you mean the number of participants per session, or the number of sessions.

 > Target selection must be randomized by using a Random Number Generator (RNG) in a computer or similar electronic device

 An RNG "in" a computer can mean a pseudorandom algorithm or a true hardware-based RNG. Clarify.

 > Randomization procedures must not be manipulated by the experimenter or participant;

 What does "manipulated" mean in this context? If I select from a table of random numbers, that is a manual process, and thus could be interpreted as manipulated.

 > *Researches In Parapsychology*

 You mean "conference proceedings such as those published in a book series called ***Research **In Parapsychology"*

*> *As standardized measure of effect size, we used that one 

 we used **the **one

 > Binomial Z score/√number of trials using the number of trials, the hits score and the chance probability as raw scores

 A comma is necessary to clarify this sentence: Binomial Z score/√number of trials **,** using the number of trials, the hits score and the chance probability as raw scores

*> *were transformed in the Hedge’s *g* effect sizes,

 were transformed into **the **Hedge’s *g* effect sizes,

 > a Bayesian random-effect model for testing its robustness

 What does "it" refer to?

 > See syntax details provided as extended data

 What does **extended data **mean? 

 In Table 2 we find a cell with the number 1909. It isn't clear what that refers to. Is that a Bayes Factor?

 > We compared the influence of the following tree moderators:

 What is a tree moderator? Do you mean **three **moderators?

 > the “file drawer” is pretty empty,

 Can you be more objective? Pretty can mean all sorts of things.

 >The results of the cumulative meta-analysis is represented 

 Results **are**, not is.

 >  Thus, it appears to be stable

 Should probably emphasize *cumulatively *stable, or stable on average.

Is the work clearly and accurately presented and does it cite the current literature?

Partly

If applicable, is the statistical analysis and its interpretation appropriate?

Yes

Are all the source data underlying the results available to ensure full reproducibility?

Yes

Is the study design appropriate and is the work technically sound?

Yes

Are the conclusions drawn adequately supported by the results?

Yes

Are sufficient details of methods and analysis provided to allow replication by others?

Partly

Reviewer Expertise:

Experimental psychology, parapsychology, psychophysiology, physics, and statistics.

I confirm that I have read this submission and believe that I have an appropriate level of expertise to confirm that it is of an acceptable scientific standard, however I have significant reservations, as outlined above.

# Response

**Published:** 2024-01-31

**Authors:** Patrizio Tressoldi

**Affiliations:** Studium Patavinum - Padova University, Italy

Sorry for the late response to your review, but we preferred to wait for further reviews' before revising the paper.

 Here are the replies to your comments.

 >My primary comment about this document is that it would be difficult for those who are not already familiar with the relevant literature to fully understand what this experiment is about. For example, in the Introduction, participants are referred to as "they," but given that "they" is sometimes used a neutral gender term, we cannot tell if this entails one person or two, who is describing their impressions aloud, how that information is recorded, who judges that information against the target, how many targets and decoys are involved (we read four at one point, and binary at another), and so on.

 Because of such confusion, I think it would be much clearer if the various types of ganzfeld condition experiments that are being considered were defined upfront, and then state how many participants are involved in each kind of study, and exactly what each of their roles are. As currently written, some of this information is provided later in the Introduction, but by the time the reader gets to that description they will already be confused.

 Reply: See restructured and corrected text in the Introduction, margin comments, and additional text.

 Other phases that might be clarified:

**Reply: We revised the introduction following these suggestions**

 What does extended data mean?

**Reply: This is an editorial requirement replacing the Supplementary Material.**

 In Table 2 we find a cell with the number 1909. It isn't clear what that refers to. Is that a Bayes Factor?

**Reply: Correct, even If after a re-analysis this value is now 85 in the range of "very strong evidence" .**

 > the “file drawer” is pretty empty,

 Can you be more objective? Pretty can mean all sorts of things.

**Reply: we deleted the term “pretty”.**
